# Characterizing Short-Time Aging Precipitation Behavior of a Novel Nickel–Iron-Based Alloy via Electrical Performance

**DOI:** 10.3390/ma17164143

**Published:** 2024-08-21

**Authors:** Junjian Cai, Chengkai Qian, Xin Huo, Qu Liu, Kejian Li, Wen Ji, Zheng Li, Zhengang Yang, Jun Cheng, Manjie Fan, Zhipeng Cai

**Affiliations:** 1 Department of Mechanical Engineering, Tsinghua University, Beijing 100084, China; caijj22@mails.tsinghua.edu.cn (J.C.); liuqu@mail.tsinghua.edu.cn (Q.L.); kejianli@mail.tsinghua.edu.cn (K.L.); 18810501261@163.com (Z.L.); yzghebut@163.com (Z.Y.); chengj22@mails.tsinghua.edu.cn (J.C.); 2 AVIC Changhe Aircraft Industry (Group) Co., Ltd., Jingdezhen 333000, China; qcknn1@gmail.com; 3 Shanghai Electric Gas Turbine Co., Ltd., Shanghai 200240, China; huoxin@shanghai-electric.com (X.H.); fanmj@shanghai-electric.com (M.F.); 4 Tianjin Research Institute for Advanced Equipment, Tsinghua University, Tianjin 300304, China; jiwen@tsinghua-tj.org

**Keywords:** nickel–iron-based alloy, short-term aging, resistivity, $\gamma^{\prime }$ resistivity

## Abstract

In this paper, the precipitation behavior and its effect on resistivity in a new type of nickel–iron-based alloy during short-term aging were investigated. During the aging process, the $\gamma^{\prime }$ phase increases in average size and decreases in number, with its area fraction fluctuating over time. This fluctuation is caused by the mismatch in the redissolution and growth rates of the $\gamma^{\prime }$ phase. As the area fraction of the $\gamma^{\prime }$ phase increases, the content of solute atoms in the matrix that scatter electrons decreases, lowering the resistivity of the alloy. Additionally, the continuous precipitation of $M_{23}C_{6}$ at grain boundaries during aging causes the resistivity to gradually increase. This paper explains the fluctuation in the total amount of $\gamma^{\prime }$ phase during short-term aging and proposes a new method for characterizing the precipitation behavior of the $\gamma^{\prime }$ phase in the novel alloy using the relative trend of resistivity changes.

## 1. Introduction

Nickel-based and iron–nickel-based superalloys are widely utilized in advanced ultra-supercritical (A-USC) steam power generation technology due to their outstanding mechanical properties at high temperatures, including tensile strength, yield strength, and creep resistance [1,2,3,4]. These properties are intrinsically linked to factors such as the size, volume fraction, and distribution of the precipitated phases [5,6,7,8,9]. As one of the common strengthening phases in nickel-based alloys, the precipitation of $\gamma^{\prime }$ phases, including Ni_3_Al and Ni_3_Ti, significantly influences the mechanical properties of nickel-based alloys. For instance, with the progression of aging, the size of the $\gamma^{\prime }$ phases gradually increases, and in certain alloys the $\gamma^{\prime }$ phases may transition from a spherical to a cubic shape. These morphological changes can significantly influence the material’s mechanical properties, including its tensile and fatigue behaviors [7,10,11,12,13,14,15,16]. Consequently, ideal properties can be achieved through the optimization of the $\gamma^{\prime }$ phase shape and distribution [17]. The alloy studied in this research is a novel $\gamma^{\prime }$ precipitation-strengthened nickel–iron-based alloy. To ensure its long-term service reliability, it is necessary to investigate the microstructural evolution of this iron–nickel-based superalloys under different heat treatment conditions.

To characterize the morphology and size evolution of the precipitates in nickel–iron-based alloys during the aging process, current research methods include direct characterization techniques such as scanning electron microscopy (SEM) [18,19] and transmission electron microscopy (TEM) [20,21], as well as indirect characterization techniques like tensile tests [22,23], impact tests [24,25], creep tests [26,27], and differential scanning calorimetry (DSC) [28]. Additionally, some researchers have used neutron diffraction to characterize the elastic lattice strains in the γ matrix, $\gamma^{\prime }$ phases, and $\gamma^{\prime \prime }$ phases during creep tests, stress relaxation tests, and stress-free aging experiments in Inconel 718 alloys [6]. However, among these precipitation characterization techniques, SEM, TEM, and DTA have higher requirements for preparation. Besides, due to the tiny sample size used in TEM, it is challenging to obtain the overall characteristics of macroscopic precipitates. Meanwhile, it is difficult to conduct large-scale mechanical and thermodynamic experiments in actual production due to their long experimental cycles. Therefore, there is an urgent need for a characterization technique that has relatively low sample preparation requirements and a short experimental cycle, while remaining sensitive to microstructural changes.

The microstructure of the alloy, including precipitates and the matrix, changes during the aging process, which in turn affects the electrical properties. Hence, the resistivity test holds significant value for characterizing the precipitation evolution during the aging process. Tian et al. [29] found that the scattering effect of Fe solute atoms in the Cu matrix is the primary cause of changes in the electrical properties of the Cu-Fe composite during aging. The precipitation of Fe-rich phases leads to a decrease in the resistivity of the composite, while the re-dissolution of Fe atoms into the matrix increases the resistivity. Short-range-ordered (SRO) structures and solute atom-enriched regions, i.e., Guinier Preston zones (GP zones), also significantly impact the electrical properties [30,31,32]. Lee, SH et al. [33] characterized the precipitation behavior of solute atoms in Al-Zn-Mg-Cu alloys during natural aging by monitoring changes in resistivity, observing a rapid rise in resistivity due to the formation of GP zones as natural aging-time increased. Zhang et al. [34] investigated the influence of Ag and Ge elements on the aging behavior of AA6061 alloys. It was found that Ge atoms more readily form secondary phases with Mg atoms, reducing the concentration of Mg atoms in the solid solution and thus the alloy’s resistivity. In the overaging stage, the resistivity increased due to the aggregation of Ag atoms and their inhibitory effect on the ordering of precipitates. These studies indicate that the resistivity tests can be used to characterize the precipitation behavior of alloys during heat treatment. However, the resistivity tests are primarily applied to conductors and metal films with few element types, such as Cu and Al, and there is limited research on the characterization of the aging behavior of complex nickel–iron-based alloys.

This paper aims to investigate the relationship between the resistivity changes and the precipitate evolution in the new type of nickel–iron-based alloy during short-term aging. Through resistivity measurements and SEM observations, the variations in resistivity, size, quantity, and distribution of $\gamma^{\prime }$ phases at different aging times were analyzed. The precipitation of $M_{23}C_{6}$ at grain boundaries was also investigated. Combining solute atom diffusion theory, the reasons for the fluctuating total precipitation amount of $\gamma^{\prime }$ phases were analyzed. Based on the insight into the precipitation behavior of nickel–iron-based alloy, a new method for determining the variation trend of $\gamma^{\prime }$ phase density during short-term aging was proposed.

## 2. Materials and Methods

### 2.1. Materials and Heat Treatment

**Figure 1:**
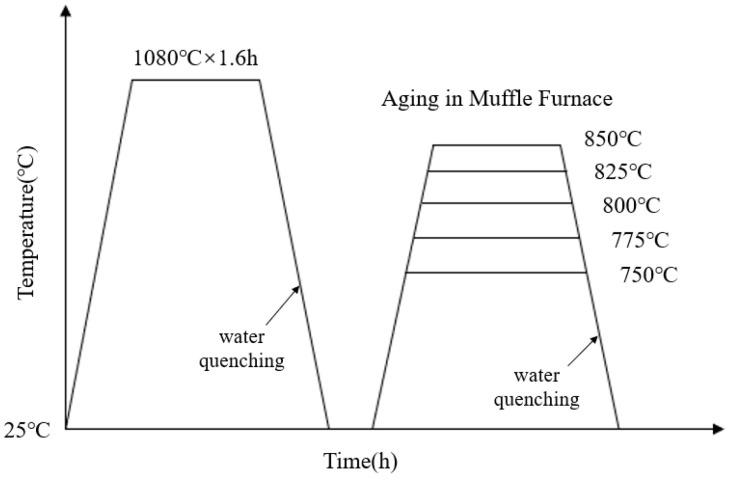
Temperature–time curve for solution and aging heat treatment.

The composition of the novel nickel–iron-based alloy was analyzed using the electron probe microanalyzer analyzer (EPMA, JXA8230, JEOL, Tokyo, Japan) with a surface scan performed at a voltage of 20 kV, as detailed in Table 1. Due to the necessity of eliminating the dendritic and precipitated phases inherent in the material before aging [9,35], a complete solution treatment was required prior to the aging process. Three 50 mm × 30 mm × 25 mm samples were solution-treated at 1080 °C. To avoid the oxidation and hardened layer, a uniform 2 mm-thickness layer was removed from all exposed sides. Then, samples were cut into 15 mm × 10 mm × 8 mm specimens. After resistivity measurement, the solution-treated specimens were placed in a muffle furnace for aging at 750 °C, 775 °C, 800 °C, 825 °C, and 850 °C for 0.5 h to 20 h. The heat treatment process is illustrated in Figure 1.

### 2.2. Electrical Resistivity Measurement

To characterize the changes in resistivity brought by aging treatment, the resistivity of each specimen was measured before and during the aging process. Before each resistivity measurement, the surfaces of the specimen were polished to become scratch-free to diminish the impact of the oxidation layer and surface roughness on the resistivity. A direct current resistance measuring instrument (QJ36, Shanghai, China) was used to measure and calculate the resistivity with the four-point method. To minimize the influence of probe-contact force on the measurement results, probes were soldered to the specimen surface using tin solder. To eliminate measurement errors caused by contact potential and thermoelectric potential, the positive and negative currents were measured 5 times, respectively, and the average value was taken as the resistivity of the sample. The relative change in resistivity was calculated using the following formula:

(1) $$\frac{∆\rho }{\rho_{0}}=\frac{\rho_{Ageing}-\rho_{0}}{\rho_{0}}$$

where $\rho_{0}$ and $\rho_{Ageing}$ represent the electrical resistivity of the same specimen in the solution-treated state and the aged state, respectively. A solution-treated specimen was selected as the control, which only underwent repeated measurements accompanying the testing specimens without the aging treatment.

### 2.3. Microstructure Characterization

(1) Scanning Electron Microscopy (SEM) Observation

Specimens were cut into 15 mm × 10 mm × 3 mm dimensions for scanning electron microscopy (SEM) observation using an SU8220 field-emission SEM (Tokyo, Japan) at 5 kV, with their surfaces polished to become scratch-free. The specimens were electrolytic etched for 30 s under a current of 1.2 A in the etching solution mixed with $65\;mL\;H_{3}{PO}_{4}+15\;mL\;H_{2}{SO}_{4}+12\;mL\;H_{8}O_{3}+3\;g\;H_{2}O+5\;g\;{CrO}_{3}$. One grain was randomly selected from five different regions on the surface. The attributes of the $\gamma^{\prime }$ phase were statistically analyzed in interior regions of each grain distant from grain boundaries and carbides. The aggregate of $\gamma^{\prime }$ particles counted in each specimen was not less than 1.3 × 10^3^. The total area of the $\gamma^{\prime }$ phase among each region was measured using ImageJ version 1.54f [36], and the total area fraction was calculated by:

(2) $$Area\;Fraction=\frac{\sum_{i=1}^{5}A_{i}}{\sum_{i=1}^{5}S_{i}}$$

where $A_{i}$ represents the total area of the $\gamma^{\prime }$ phase in region i, and $S_{i}$ represents the total area of region i.

(2) Electron Backscatter Diffraction (EBSD) Tests

EBSD tests were used to assess the effect of aging treatment on the microstructure. Specimens aged at 850 °C for 1 h and 10 h were mechanically polished, followed by ion-beam milling with an ion-beam mill (Leica EM TIC 3X, Wetzlar, Germany). During milling, the argon ion beam was accelerated at 5.5 kV with a current of 1.5 mA for a duration of 2.5 h. EBSD tests were conducted using a scanning electron microscope (TESCAN S9000X, Brno, Czech Republic) equipped with an EBSD detector. The electron beam was accelerated at 15 kV. A 250 μm × 250 μm region was scanned for phase distribution with a step size of 0.5 μm, and a 10 μm × 10 μm region was scanned in detail with a step size of 0.025 μm.

(3) X-ray Diffraction (XRD) Analysis

XRD analysis was performed on the specimens aged at 850 °C using an X-ray diffractometer (Rigaku D/max-2500 diffractometer, Tokyo, Japan) with a scanning rate of 1°/min from 2θ = 40° to 125°. A Cu target with its Kα1 wavelength equal to 1.542 Å was employed. A Cu target was employed with its Kα1 wavelength equal to 1.542 Å.

## 3. Results

### 3.1. Resistivity Changes Induced by Aging Treatment

**Figure 2:**
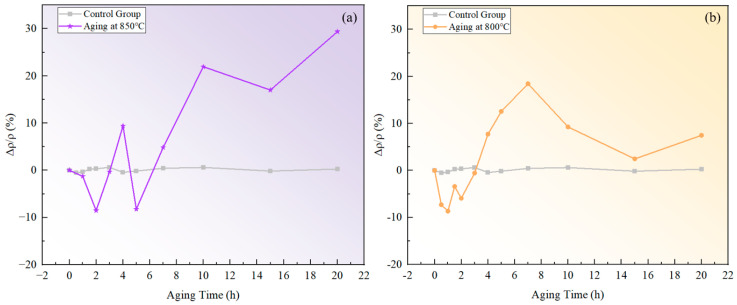
Relative changes in resistivity at different temperatures over aging times: (**a**) 850 °C; (**b**) 800 °C.

The measured resistivity of the novel nickel–iron-based alloy in the solution-treated state was approximately 55.36±4.59 μΩ·cm. The resistivities of both specimens aged at 800 °C and 850 °C exhibited a fluctuating pattern in the $∆\rho /\rho_{0}$ over time with a general upward trend as time increased, as illustrated in Figure 2. In comparison, the $∆\rho /\rho_{0}$ of the control specimen varied within a tiny scale from −0.53% to 0.62%, indicating a fine accuracy and reliability of the resistivity measurement.

### 3.2. Evolution of γ’ Precipitate Morphology

**Figure 3:**
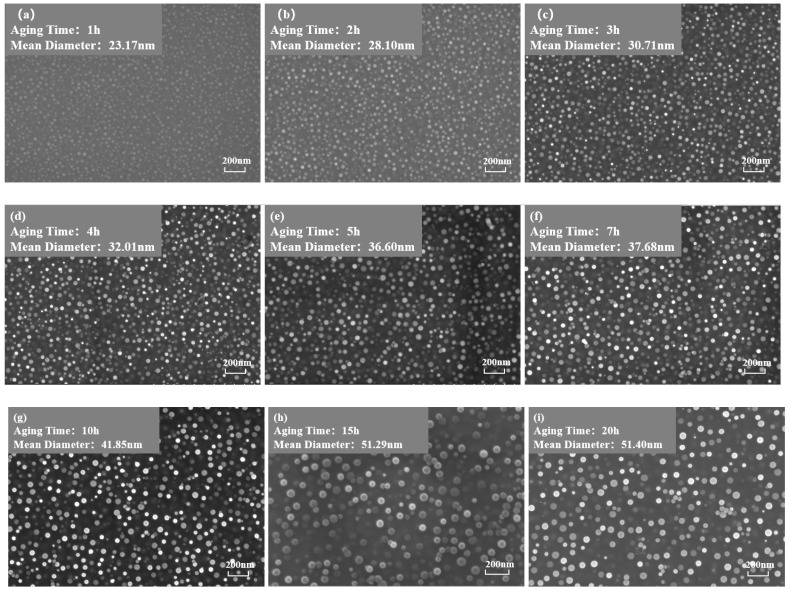
Morphology of the $\gamma^{\prime }$ phase after aging at 850 °C for different durations: (**a**) 1 h, (**b**) 2 h, (**c**) 3 h, (**d**) 4 h, (**e**) 5 h, (**f**) 7 h, (**g**) 10 h, (**h**) 15 h, (**i**) 20 h.

**Figure 4:**
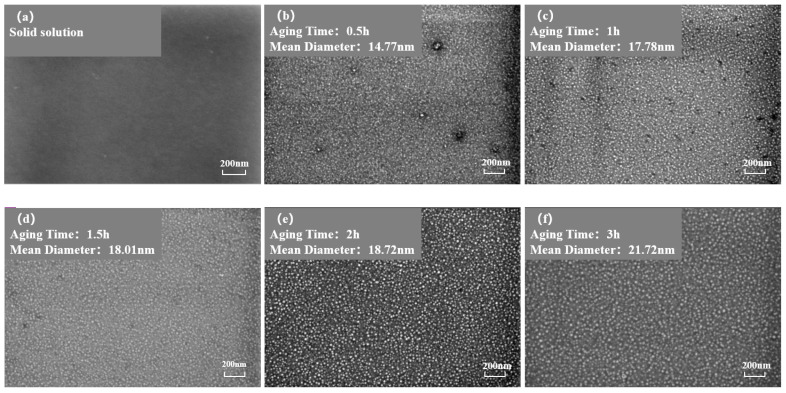

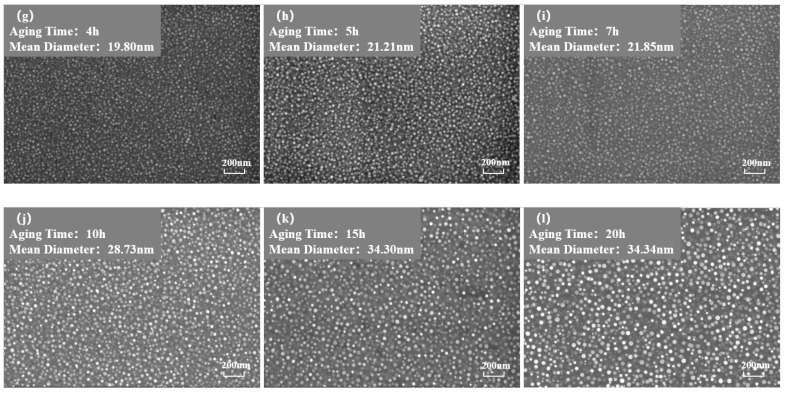
Morphology of the $\gamma^{\prime }$ phase after aging at 850 °C different durations: (**a**) 0 h, (**b**) 0.5 h, (**c**) 1 h, (**d**) 1.5 h, (**e**) 2 h, (**f**) 3 h, (**g**) 4 h, (**h**) 5 h, (**i**) 7 h, (**j**) 10 h, (**k**) 15 h, (**l**) 20 h.

**Figure 5:**
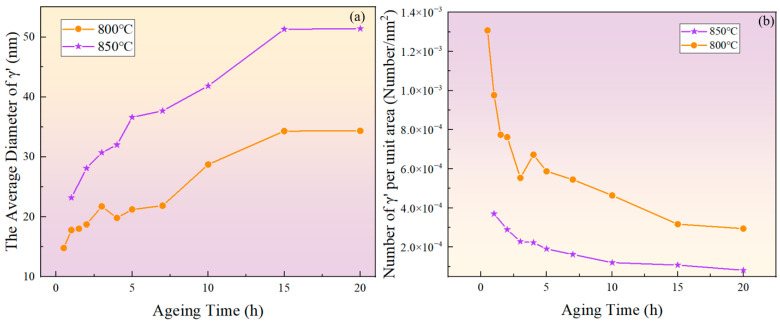
(**a**) Evolution of the average diameter of the $\gamma^{\prime }$ phase with aging time at 800 °C and 850 °C; (**b**) evolution of particle density with aging time at 800 °C and 850 °C.

Figure 3 and Figure 4 show the morphology of the $\gamma^{\prime }$ phase in the solution-treated alloy after aging at 850 °C and 800 °C over time, respectively, and the average diameter of the $\gamma^{\prime }$ phase is given in Figure 5a. The results indicate that, after aging for the same duration, the particle diameters at 850 °C were larger than those at 800 °C. During aging from 1 h to 20 h at 850 °C, the average particle radius of the $\gamma^{\prime }$ phase increased from 23.17 nm to 51.40 nm. At 800 °C, the aging process from 0.5 h to 20 h saw the average particle radius of the $\gamma^{\prime }$ phase grow from 14.77 nm to 34.34 nm. The $\gamma^{\prime }$ phase maintained a spherical morphology throughout the aging process at both 850 °C and 800 °C.

As shown in Figure 5b, the number of $\gamma^{\prime }$ phases per unit area showed an overall decreasing trend. At the same aging duration, the number of $\gamma^{\prime }$ phases per unit area at 800 °C was significantly higher than at 850 °C. This is because at higher temperatures, the diffusion rates of solute atoms and vacancies are greater, resulting in a faster dissolution rate of smaller-sized $\gamma^{\prime }$ phases. Therefore, the higher the temperature, the lower the particle density at the same aging time.

**Figure 6:**
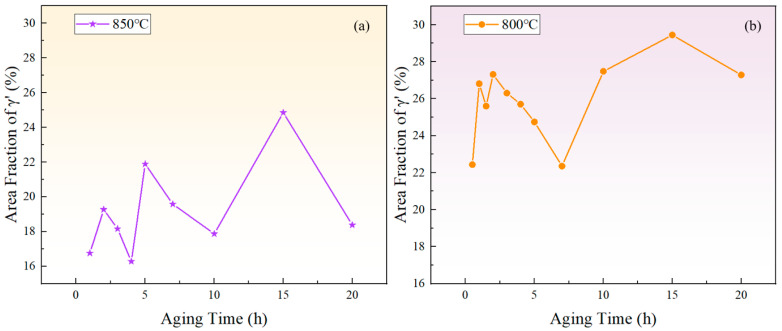
The variation of the $\gamma^{\prime }$ phase area fraction with time during short-term aging at (**a**) 850 °C and (**b**) 800 °C.

Figure 6 depicts the relationship between the area fraction of the $\gamma^{\prime }$ phase and aging time at 850 °C and 800 °C. The area fraction of the $\gamma^{\prime }$ phase fluctuates with aging time, indicating that the precipitated phase does not reach equilibrium during short-term aging. Considering the increase in the particle size and the overall decrease in the particle number over time, it can be inferred that that both precipitation and Ostwald ripening phenomena occurred during the short-term aging process. In other words, smaller particles dissolved, releasing solute atoms that then re-precipitate onto larger particles, which leads to an increase in the average particle size. 

### 3.3. Evolution of Carbide with Aging Time

**Figure 7:**
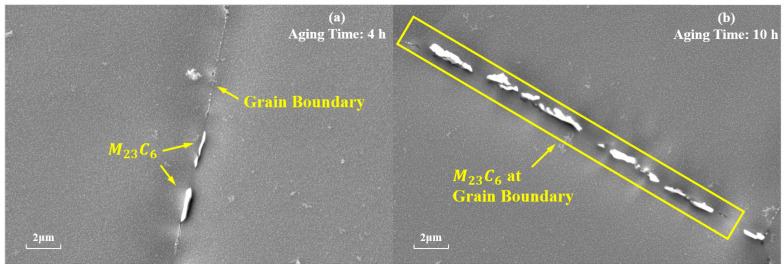
Evolution of $M_{23}C_{6}$ at the grain boundaries during aging at 850 °C for different durations: (**a**) 4 h; (**b**) 10 h.

**Figure 8:**
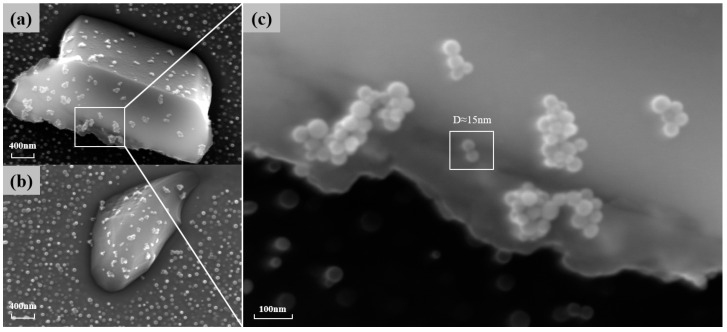
(**a**,**b**) The $\gamma^{\prime }$ particles around the MC carbide when carbide decomposition occurs at 850° C for 10 h of aging; (**c**) a magnified image of finer $\gamma^{\prime }$ particles.

$M_{23}C_{6}$ is also one of the main strengthening phases of the novel nickel–iron-based alloy. As shown in Figure 7, the precipitation of $M_{23}C_{6}$ at the grain boundaries gradually increases in number and size with the aging time at 850 °C. The morphology of these carbides also transformed from a particulate to a continuous cellular morphology along the grain boundaries. Additionally, the decomposition of the carbide ($MC+\gamma \to M_{23}C_{6}+\gamma^{\prime }$) was observed at 850 °C for 4 h and 10 h, which is consistent with phenomena observed by other researchers [8,9,37]. Consequently, finer $\gamma^{\prime }$ particles were formed around the MC carbides. As shown in Figure 8, when aged at 850 °C for 10 h, the size of $\gamma^{\prime }$ particles near MC was 15 nm, whereas the average size of $\gamma^{\prime }$ particles distant from grain boundaries and MC was 41.85 nm.

## 4. Discussion

### 4.1. Causes of the Resistivity Fluctuation

Several factors can influence electrical properties of the alloy, including the grain size, grain boundary behavior, dislocations, and precipitates [38]. In Huang’s research on the HT700P alloy, no significant change in grain morphology and size was found during the aging process [9]. Unlike cubic $\gamma^{\prime }$ phases, spherical $\gamma^{\prime }$ phases exhibit a smaller lattice misfit with the matrix [39], thus the scattering effect of phase boundaries on electrons can be disregarded. G. Kelekanjeri’s study on Waspaloy alloy suggested that, in the maturation stage, the influence of dislocations, vacancies, and other factors on resistivity was subtle compared with the solute concentration in the matrix [40,41]. The solute concentration in the matrix is closely related to precipitation behavior. Therefore, the fluctuation and upward trend of the $∆\rho /\rho_{0}-t$ curve during the aging process of the novel nickel–iron-based alloy can be explained from two aspects, namely the precipitation behavior and grain boundary behavior.

Roebuck’s study [42] on CMSX4 single-crystal, nickel-based alloy shows that the resistivity of the precipitated $\gamma^{\prime }$ phase is higher than that of the γ matrix, resulting in a positive correlation between the resistivity of this alloy and the change in the total amount of precipitation [43]. However, the resistivity of the alloy in the solution-treated state was measured to be 55.36±4.59 μΩ·cm, and studies have shown that the resistivity of the $\gamma^{\prime }$ phase is about 44.2±0.5 μΩ·cm [44], which is lower than that of the γ phase. Additionally, change in the volume fraction of the precipitates should also be considered. The precipitation of the $\gamma^{\prime }$ phase can decrease the solute concentration in the γ phase. The relationship between the resistivity of the γ phase and the solute concentration can be illustrated by the following equation [45,46,47]:

(3) $$\rho_{\gamma }=\rho_{0}+\rho_{imp}=\rho_{0}+x_{imp}\frac{∆\rho_{imp}}{a}$$

where $\rho_{0}$ is the resistivity of the alloy matrix without solute atoms, $\rho_{imp}$ is the resistivity caused by the solid solution atoms, $x_{imp}$ is the atomic fraction of the solute element, and $∆\rho_{imp}/a$ represents the change in resistivity caused by a sole solute atom. This indicates that the resistivity of the γ phase is positively correlated with the solute concentration. As a result, the precipitation of $\gamma^{\prime }$ phases can decrease the overall resistivity of the alloy.

**Figure 9:**
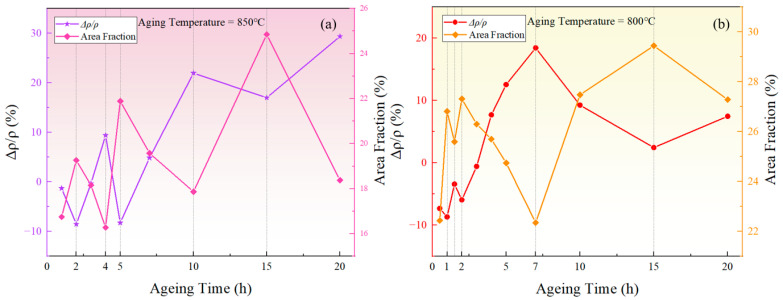
The relationship between the relative change rate of resistivity and the area fraction of the precipitated $\gamma^{\prime }$ phase during aging at (**a**) 850 °C, (**b**) 800 °C.

Taking the fluctuation of resistivity and the fluctuation of $\gamma^{\prime }$ phase area fraction at 850 °C as an example, the corresponding relationship between resistivity and area fraction fluctuations can be analyzed based on Figure 9a.

(1) During the stages of 2–4 h, 5–10 h, and 15–20 h, the area fraction of the $\gamma^{\prime }$ phase decreased, leading to the redissolution of solute atoms into the matrix. Hence, the solute concentration in the matrix increased. On one hand, the reduction in the total amount of $\gamma^{\prime }$ phases, which have lower resistivity, resulted in an overall increase in resistivity. On the other hand, the outer electrons of Al and Ti atoms tended to overlap with the 3d orbitals of transition elements including Ni, Cr, and Fe, and the redistribution of the electrons could form short-range-ordered structures similar to intermetallic compounds [48], increasing the resistivity of the alloy [30]. Additionally, the increased solute concentration in the matrix led to a greater lattice distortion, disrupting the periodicity of electron scattering, thus hindering electron scattering and increasing the resistivity.

(2) During the stages of 1–2 h, 4–5 h, and 10–15 h, the area fraction of the $\gamma^{\prime }$ phase increased. The increased amount of $\gamma^{\prime }$ phases led to an overall reduction in resistivity. In addition, the decrease in solute concentration within the matrix and the reduction in lattice distortion helped restore the periodicity of the lattice in the matrix. According to Bloch’s theorem, the movement of electrons will experience reduced energy consumption over a larger lattice range, thereby lowering the resistivity.

**Figure 10:**
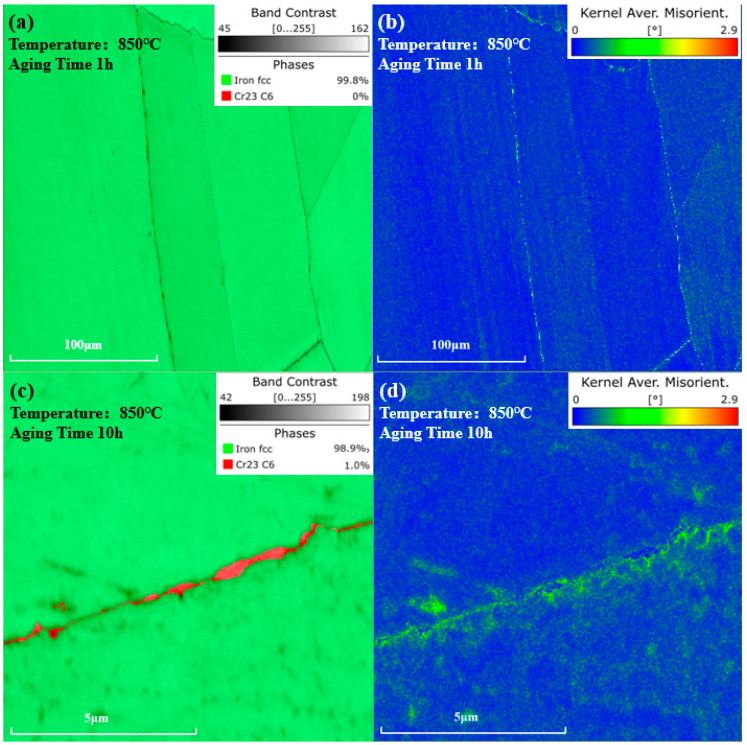
Phase and KAM value distribution maps after aging at 850 °C. (**a**) Phase map and (**b**) KAM value distribution map of the same location after 1 h of aging. (**c**) Phase map and (**d**) KAM value distribution map near the grain boundary after 10 h of aging.

As for the upward trend in resistivity, research has shown that the pinning of dislocations by $M_{23}C_{6}$ carbides leads to the enrichment of carbon atoms, thereby increasing the resistivity [49]. The kernel average misorientation (KAM) value from EBSD tests is deemed to be positively correlated with the dislocation density [50]. To be specific, the geometrically necessary dislocation (GND) density of the sample can be calculated using the following formula [51]:

(4) $$\rho_{GND}=\frac{2\theta_{KAM}}{b\cdot ∆d}$$

where b represents the Burgers vector. ∆d is the scanning step size. $\theta_{KAM}$ is the KAM angle. As shown in Figure 10a,b, after being aged at 850 °C for 1 h, no $M_{23}C_{6}$ phase was observed in the phase map, and the KAM value near the grain boundaries distributed uniformly. However, as shown in Figure 10c,d, after 10 h of aging, ${Cr}_{23}C_{6}$ carbides precipitated at grain boundaries. Higher KAM values could be observed near the carbides, indicating a greater dislocation density and higher degree of lattice distortion around the carbides, compared to other regions.

The precipitation of the $\gamma^{\prime }$ phase can cause lattice contraction, facilitating the dislocation multiplication. Strengthening phases such as $M_{23}C_{6}$ carbides can pin these dislocations. As shown in Figure 7, as aging time was prolonged, there was an increasing amount of $M_{23}C_{6}$ precipitates at the grain boundaries, leading to an increased number of pinned dislocations at these sites. This resulted in severe lattice distortion around the $M_{23}C_{6}$ phases, attracting more solute atoms and causing an increase in resistivity at the grain boundaries.

### 4.2. The Short-Term Aging Coarsening Kinetics of the $\gamma^{\prime }$ Phase

**Figure 11:**
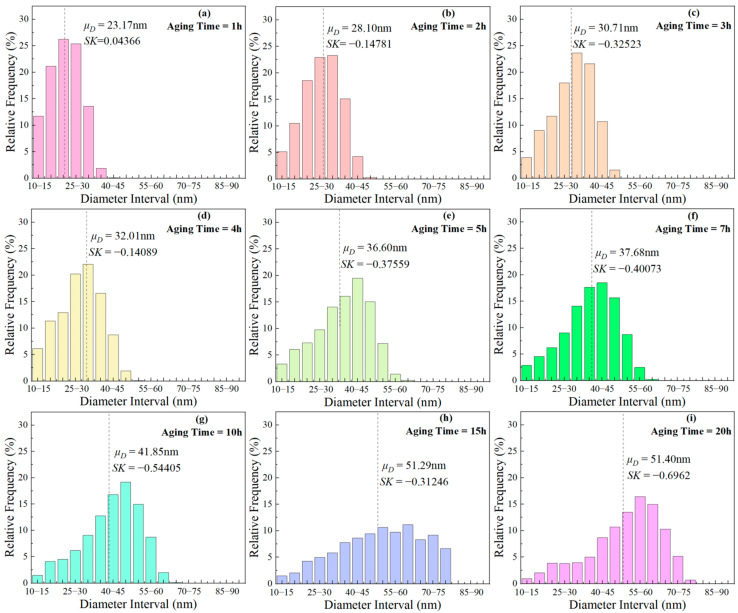
Frequency distribution histograms of $\gamma^{\prime }$ phases with different sizes after aging at 850 °C for various durations: (**a**) 1 h, (**b**) 2 h, (**c**) 3 h, (**d**) 4 h, (**e**) 5 h, (**f**) 7 h, (**g**) 10 h, (**h**) 15 h, (**i**) 20 h.

The frequency distribution histograms for $\gamma^{\prime }$ phases of different sizes during aging at 850 °C are presented in Figure 11. The calculation formula for the skewness coefficient (*SK*) is as follows:

(5) $$SK=\frac{\bar{\mu_{D}}-M}{\sigma }$$

where, $\bar{\mu_{D}}$ denotes the mean of the sample, M represents the median of the sample, and σ signifies standard deviation of the sample. When *SK* = 0, the particle diameter follows a normal distribution; *SK* > 0 indicates a higher proportion of larger-sized $\gamma^{\prime }$ phases, and vice versa. With the aging time prolongation, *SK* changed from positive to negative, indicating that small size $\gamma^{\prime }$ particles gradually dissolved, and the proportion of larger-sized $\gamma^{\prime }$ particles increased. This indicates that ripening occurs during short-term aging.

**Figure 12:**
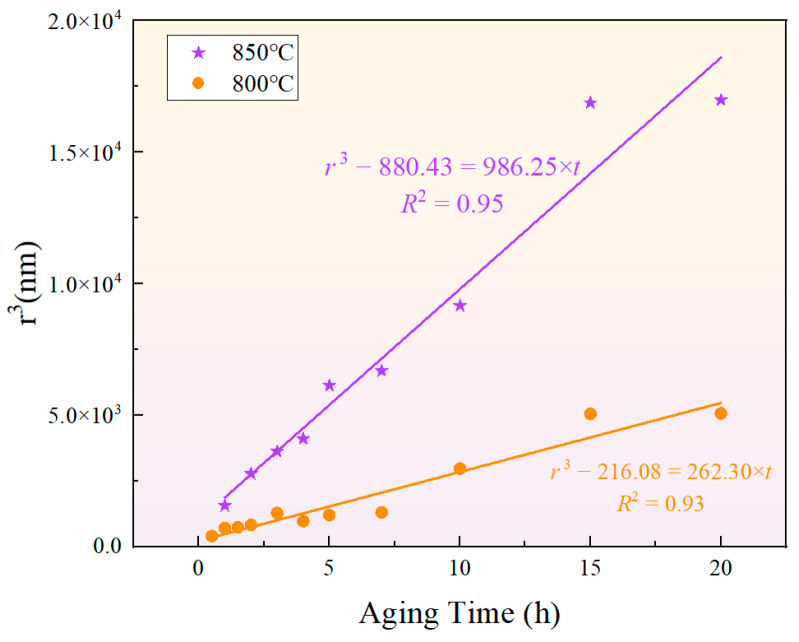
Fitting results of the relationship between $\gamma^{\prime }$ phase particle size and aging time according to the LSW ripening theory at 850 °C and 800 °C.

The classical formula for particle size evolution based on the LSW theory is given by ${\bar{r}}^{3}-r_{0}^{3}=kt$, where $\bar{r}$ represents the average radius of the $\gamma^{\prime }$ phase, $r_{0}$ is the critical nucleus radius, and k is the coarsening rate constant. Although the classical LSW theory assumes that the precipitated phase is in equilibrium [52,53,54,55], the results indicate that the cube of the $\gamma^{\prime }$ phase radius presents a relatively high fitting goodness, as shown in Figure 12. This suggests that the particle size of the $\gamma^{\prime }$ phase during short-term aging can be predicted by the LSW theory. Meanwhile, the growth rates of the $\gamma^{\prime }$ phase during short-term aging at 850 °C and 800 °C were $986.25\;{nm}^{3}/h$ and $262.30\;{nm}^{3}/h$, respectively, showing that a higher temperature results in a greater growth rate.

The activation energy for the growth of the $\gamma^{\prime }$ phase can be calculated using the equation $ln\left(kT\right)=C-Q/RT$, where k is the growth rate, T is the absolute temperature, C is a constant, and R=8.314 J/(mol·K) is the ideal gas constant. The calculated coarsening activation energy for the $\gamma^{\prime }$ phase was 277.59 kJ/mol. In the Ni matrix, the diffusion activation energy of Al was approximately 260 kJ/mol [56]~269.2 kJ/mol [57], and that of Ti in the Ni matrix was 257 kJ/mol [58,59]. Meanwhile, the diffusion activation energies of Ni and Fe in the Ni matrix were 284 kJ/mol [60,61] and 281 kJ/mol [60], respectively. Therefore, the solid-state diffusion capabilities of Al and Ti atoms are greater than those of Fe and Ni atoms. In this new type of nickel–iron-based superalloy, the coarsening activation energy of the $\gamma^{\prime }$ phase is slightly higher than that of Al and Ti, but lower than that of Ni and Fe. Therefore, under the given temperature and time conditions, the bulk diffusion of Al and Ti elements is more feasible, indicating that the growth of the $\gamma^{\prime }$ phase was primarily achieved through the bulk diffusion of Al and Ti elements, and the coarsening activation energy was dominated by the mole fractions of Al and Ti atoms in the matrix.

### 4.3. Analysis of the Fluctuation in the Total Amount of $\gamma^{\prime }$ Phase Precipitation

The transformation process of $\gamma^{\prime }$ phase is driven by the minimization of Gibbs free energy. In this process, the driving force is the difference in chemical free energy between the matrix and $\gamma^{\prime }$ phases, and the resistance is the interfacial energy and elastic strain energy. The fluctuations in the area fraction of the $\gamma^{\prime }$ phases are inherently the result of the competition between the driving and resisting forces of the phase transformation. The competition results in the rates of precipitation, redissolution, and growth during the aging process not being stable and being in competition with each other.

In the first stage of aging, the chemical free energy of the $\gamma^{\prime }$ phase was lower than that of the γ matrix. To minimize the Gibbs free energy, the $\gamma^{\prime }$ phase began to precipitate and grow, reducing the solute concentration in the matrix and increasing the total amount of the $\gamma^{\prime }$ phase. Ostwald ripening occurred in the second stage of aging. To minimize the total interfacial energy, the solute concentration at the surfaces of different particles varied due to differences in chemical potential, leading to strong interactions between $\gamma^{\prime }$ phases of different sizes. The interactions between particles involved the dissolution of smaller-sized $\gamma^{\prime }$ phases into the matrix, as well as the growth of larger-sized $\gamma^{\prime }$ phases via absorbing solute atoms from the matrix. However, the rates of two such processes may differ, which mainly accounts for the fluctuation in the area fraction during the aging process.

To explain the changes in solute concentration within the matrix, two types of equilibrium concentrations were first defined: ➀ At a specific moment, $t_{0}$, during a very short aging period, i.e., when ${\left.∆t\right|}_{t=t_{0}}\to 0$, the solute concentration at a specific site in the matrix remained almost constant during the exchange of solute atoms between the $\gamma^{\prime }$ particles and the matrix. This concentration is defined as $C_{e}$, representing the equilibrium solute concentration in the matrix. ➁ When the aging time t→∞, the precipitation process reached equilibrium, and the equilibrium concentration of solute atoms in the matrix at this point is defined as $C_{\infty }$. The following assumptions are made specific to the short-term aging: ➀ the distribution of $\gamma^{\prime }$ phases in the matrix was random; ➁ the equilibrium concentration of solute atoms, $C_{e}$, was equal among all particles in the matrix.

(1) Stage I: the precipitation and growth of the $\gamma^{\prime }$ phase.

**Figure 13:**
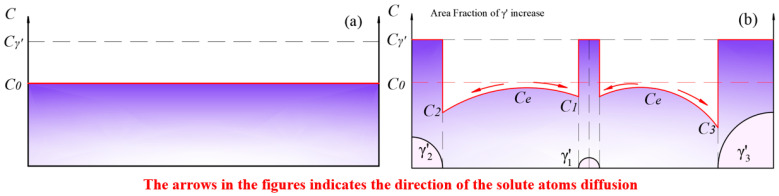
(**a**) The solid solution matrix with a solute concentration of $C_{0}$; (**b**) the precipitation and growth stages of the $\gamma^{\prime }$ phase.

The solute concentration distribution within the matrix in the solid solution state during the initial stage of aging is depicted in Figure 13. In this stage, $\gamma^{\prime }$ phases precipitated and grew rapidly. At this point, the equilibrium concentration $C_{e}$ within the γ matrix was higher than the solute concentration in the matrix near the surface of the $\gamma^{\prime }$ phase. Solute atoms diffused from regions of high concentration to the surface of the $\gamma^{\prime }$ phase with lower concentration, causing a decrease in the solute concentration in the matrix. The growth of the $\gamma^{\prime }$ phase led to an increase in the area fraction.

(2) Stage II: the redissolution of smaller-sized $\gamma^{\prime }$ particles and growth of larger-sized $\gamma^{\prime }$ particles.

**Figure 14:**
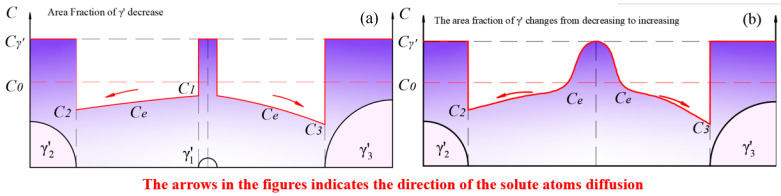
Solute concentration distribution at (**a**) the stage when the redissolution process of smaller-sized $\gamma^{\prime }$ particles was dominant; (**b**) the moment when the smaller-sized $\gamma^{\prime }$ phase disappeared.

As shown in Figure 14a, when the equilibrium concentration $C_{e}$ in the matrix decreased to $C_{2},\;C_{3}<C_{e}<C_{1}$, solute atoms at the surface of smaller-sized $\gamma^{\prime }$ particles dissolved back into the matrix, while larger-sized $\gamma^{\prime }$ particles continued to grow. To compare the rates of redissolution and growth, the following definitions are made: the radius of the smaller-sized $\gamma^{\prime }$ particles is $r_{S}$, the solute atom concentration in the matrix near the surface of the smaller-sized particles is $C_{S}$, the radius of the larger-sized $\gamma^{\prime }$ particles is $r_{B}$, and the solute atom concentration in the matrix near the surface of the larger-sized $\gamma^{\prime }$ particles is $C_{B}$. Since the driving force for Ostwald ripening is the difference in chemical potential between the smaller-sized and larger-sized $\gamma^{\prime }$ particles, the chemical potential at the equilibrium concentration, $C_{e}$, is defined as $\mu_{e}$, the chemical potential of solute atoms near the surface of the larger-sized $\gamma^{\prime }$ particles in the matrix is defined as $\mu_{B}$, and the chemical potential of solute atoms near the surface of the smaller-sized $\gamma^{\prime }$ particles in the matrix is defined as $\mu_{S}$. According to Raoult’s law:

(6) $$\mu_{i}=\mu_{i}^{0}+RTlnC_{i}$$

where $\mu_{i}^{0}$ is the chemical potential of component i at a concentration of 100%, R=8.314 J/(mol·K) is the ideal gas constant, T is the absolute temperature, and $C_{i}$ is the concentration of component i.

We assume that the chemical driving force for the dissolution of small-sized $\gamma^{\prime }$ and the growth of large-sized $\gamma^{\prime }$ is identical, i.e., $\mu_{S}-\mu_{e}=\mu_{e}-\mu_{B}$. In this case, the equilibrium concentration between the two particles is $C_{e}=\sqrt{C_{S}\cdot C_{B}}$.

According to the spherical symmetry steady-state diffusion equation:

(7) $$\frac{dm_{i}}{dt}=4\pi D_{i}r_{i}\left(C_{e}-C_{i}\right)$$

(8) $$\frac{1}{\Omega }\cdot 4\pi r_{i}^{2}\cdot \frac{dr_{i}}{dt}=4\pi D_{i}r_{i}\left(C_{e}-C_{i}\right)$$

where $\frac{dm_{i}}{dt}$ is the flow rate of solute atoms through the spherical shell of the $\gamma^{\prime }$ particle with a radius of $r_{i}$ due to diffusion, Ω is the volume of the new phase formed by a mole of solute atoms, and $D_{i}$ is the diffusion coefficient of solute atoms in the matrix near the surface of the $\gamma^{\prime }$ particle with a radius of $r_{i}$. Since $\frac{dA}{dt}=2\pi r\cdot \frac{dr}{dt}$, Equation (8) can be transformed into:

(9) $$2\frac{r_{i}}{\Omega }\cdot \frac{dA_{i}}{dt}=4\pi D_{i}r_{i}\left(C_{e}-C_{i}\right)$$

(10) $$\frac{dA_{i}}{dt}=2\pi \Omega D_{i}\left(C_{e}-C_{i}\right)$$

Considering that the concentration at the particle surface is $C_{i}=C_{\infty }\left(1+\frac{2\gamma \Omega }{RTr_{i}}\right)$ [62], the smaller the radius of the $\gamma^{\prime }$ particle, the higher the solute concentration is in the surrounding matrix, i.e., $C_{B}<C_{e}<C_{S}$. Combining Equation (10), it can be found that $\frac{dA_{B}}{dt}>0$, indicating that the larger-sized $\gamma^{\prime }$ particles continue to grow, and $\frac{dA_{S}}{dt}<0$, indicating that the smaller-sized $\gamma^{\prime }$ particles will dissolve back into the matrix. The total area change of the large and small particles during the competitive growth process is given by:

(11) $$\frac{dA}{dt}=\frac{dA_{B}}{dt}+\frac{dA_{S}}{dt}=2\pi \Omega \left(\sqrt{C_{S}}-\sqrt{C_{B}}\right)\left(D_{B}\sqrt{C_{B}}-D_{S}\sqrt{C_{S}}\right)$$

The diffusion coefficient, $D_{i}$, of solute atoms in the matrix is concentration-dependent. Within the temperature range of 800 °C to 900 °C, when the Al content in the Fe-based matrix was less than 20%, the interdiffusion coefficient D increased with the concentration of Al solute atoms [63]. Similarly, the diffusion coefficient of Al in Ni-based matrices also increased with the concentration of Al [64]. This implies that $D_{S}>D_{B}$ and $D_{B}\sqrt{C_{B}}-D_{S}\sqrt{C_{S}}<0$. That is, for two adjacent $\gamma^{\prime }$ particles of different sizes, when competitive growth occurs, it can be found from Equation (11) that $\frac{dA}{dt}<0$, meaning that when competitive growth occurs, the dissolution rate of the smaller-sized $\gamma^{\prime }$ exceeds the growth rate of the larger-sized $\gamma^{\prime }$ under the same chemical driving force. As a result, the dissolution of the smaller-sized $\gamma^{\prime }$ dominated at this stage, leading to a decrease in the area fraction. Such a process maintained until the moment when the smaller-sized $\gamma^{\prime }$ phase disappeared, as shown in Figure 14b. The dissolution rate fell to 0, while residual solute atoms kept contributing to the growth of the adjacent $\gamma^{\prime }$ phase. In other words, Figure 14b represents the inflection point where the area fraction changed from decreasing to increasing.

(3) Stage III: a new loop of growth and redissolution process of the $\gamma^{\prime }$ phase.

**Figure 15:**
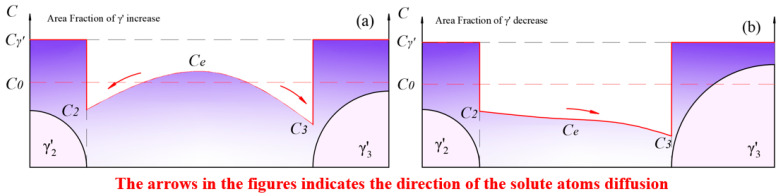
The new round of fluctuation in the total amount of $\gamma^{\prime }$ phase: (**a**) the growth process of $\gamma^{\prime }$ phase dominates in the new loop; (**b**) the redissolution process of $\gamma^{\prime }$ phase dominates in the new loop.

As shown in Figure 14b, with the complete dissolution of the smaller-sized $\gamma^{\prime }$ particles, the interaction between adjacent $\gamma^{\prime }$ particles transformed from ${\gamma^{\prime }}_{1}↔{\gamma^{\prime }}_{2}$ and ${\gamma^{\prime }}_{1}↔{\gamma^{\prime }}_{3}$ to ${\gamma^{\prime }}_{2}↔{\gamma^{\prime }}_{3}$. At this point, the solute concentration in the matrix reached a maximum. Subsequently, the process shown in Figure 15a began, where solute atoms diffused from high-concentration areas to low-concentration areas. The growth of $\gamma^{\prime }$ phases led to an increase in the area fraction and a decrease in the solute concentration in the matrix.

After a period of aging, the equilibrium concentration, $C_{e}$, in the matrix further decreased to $C_{3}<C_{e}<C_{2}$, when a new loop of competitive growth occurred. The redissolution rate was still greater than the growth rate, leading to a continuous fluctuation in the area fraction.

In summary, during aging for 1–2 h, 4–5 h, and 10–15 h at 850 °C, and during aging for 0.5–1 h, 1.5–2 h, and 7–15 h at 800 °C, the area fraction showed an upward trend, indicating that the growth of the larger-sized $\gamma^{\prime }$ phase dominated in these periods, and the solute concentration in the matrix decreased. In contrast, during aging for 2–4 h, 5–10 h, and 15–20 h at 850 °C, and during aging for 1–1.5 h, 2–7 h, and 15–20 h at 800 °C, the area fraction exhibited a downward trend, suggesting that the redissolution of the smaller-sized $\gamma^{\prime }$ phase was the predominant process, and the solute concentration in the matrix increased.

**Figure 16:**
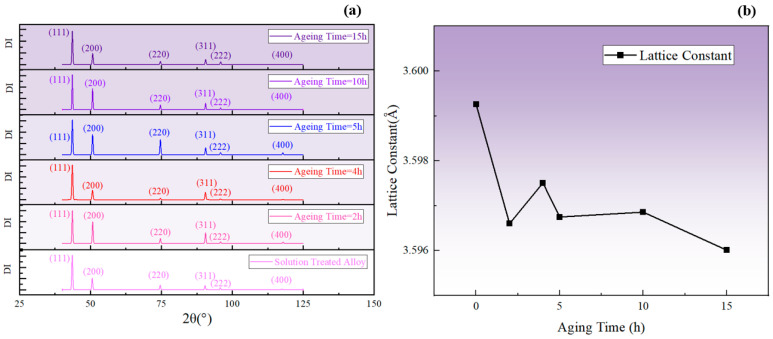
(**a**) XRD diffraction patterns corresponding to different aging times at 850 °C; (**b**) changes in the average lattice constant calculated from 2*θ*.

It is known that the lattice constant of the $\gamma^{\prime }$ phase was smaller compared to the γ matrix. Additionally, the decrease in solute atom concentration in the γ matrix led to a reduction in the degree of lattice distortion in the γ matrix. Therefore, when the total amount of the $\gamma^{\prime }$ phase increased, the lattice constant of the alloy decreased, corresponding to a shift in the diffraction peak to a higher degree, and vice versa. The change in the average lattice constant can be taken as a reflection of the fluctuation in the total amount of the $\gamma^{\prime }$ phase and, as shown in Figure 16, the lattice constant increased during aging for 2–4 h and 5–10 h at 850 °C, corresponding to a decrease in the total amount of the $\gamma^{\prime }$ phase. In contrast, the lattice constant decreased during aging for 0–2 h, 4–5 h, and 10–15 h, indicating an increase in the total amount of the $\gamma^{\prime }$ phase. The fluctuation in the lattice constant obtained by XRD was consistent with the SEM results and the fluctuation pattern of resistivity.

### 4.4. Characterization of Resistivity and Precipitation during Aging at Other Temperatures

**Figure 17:**
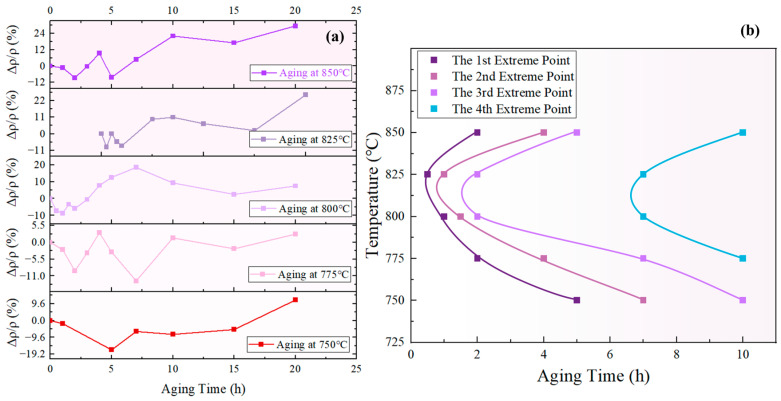
(**a**) $∆\rho /\rho_{0}-t$ curves at different temperatures; (**b**) extreme curve of total $\gamma^{\prime }$ phase content using resistivity characterization.

According to the prior analysis, an increase in $∆\rho /\rho_{0}$ indicates that the redissolution of small-sized $\gamma^{\prime }$ particles was the dominant process, leading to a decrease in the total amount of the $\gamma^{\prime }$ phase. Conversely, a decrease in $∆\rho /\rho_{0}$ suggests that the growth of the $\gamma^{\prime }$ phase is the dominant process, resulting in an increase in the total amount of the $\gamma^{\prime }$ phase. Therefore, the inflection points in the $∆\rho /\rho_{0}-t$ curve can be regarded as the transition points between alternate dominance of the redissolution and growth processes. Figure 17a displays the $∆\rho /\rho_{0}-t$ curves of the novel nickel–iron-based alloy aged at 750 °C, 775 °C, 800 °C, 825 °C, and 850 °C for various durations, which all exhibited fluctuation. Four curves can be obtained by connecting the 1st, 2nd, 3rd, and 4th extreme values in order of temperature, as shown in Figure 17b.

The curve formed by the first and third minimum points of the $∆\rho /\rho_{0}-t$ curve in Figure 17a is defined as the “maximum total content of $\gamma^{\prime }$ phase curve”, corresponding to the first and third curves from the left in Figure 17b. Likewise, the curve formed by the second and fourth maximum points of the $∆\rho /\rho_{0}-t$ curve in Figure 17a is defined as the “minimum total content of $\gamma^{\prime }$ phase curve”, corresponding to the second and fourth curves from the left in Figure 17b. These curves can be used to determine whether the total content of the $\gamma^{\prime }$ phase increases or decreases during a specific aging period at the given temperature. For example, at 825 °C the total content of the $\gamma^{\prime }$ phase decreased during aging for 0.5–1 h, increased during aging for 1–2 h, and then decreased again during aging for 2–7 h.

As observed in Figure 17b, there is a general trend of increasing interval between the extrema curves of the total content of the $\gamma^{\prime }$ phase. In line with the prior analysis, the fluctuation in the total volume of the $\gamma^{\prime }$ phase is attributed to the alternating dominance of the redissolution process of small-sized $\gamma^{\prime }$ phases and the growth process of large-sized $\gamma^{\prime }$ phases, both of which were driven by the bulk diffusion of solute elements. Consequently, the interval between the extrema curves is associated with the time taken for solute atom concentrations to reach equilibrium. According to Fick’s Second Law, $\frac{\partial C\left(x,t\right)}{\partial t}=D\frac{\partial^{2}C\left(x,t\right)}{\partial x^{2}}$, it is evident that the concentration distribution of solute elements in the matrix was influenced by both time and co-ordinates.

**Figure 18:**
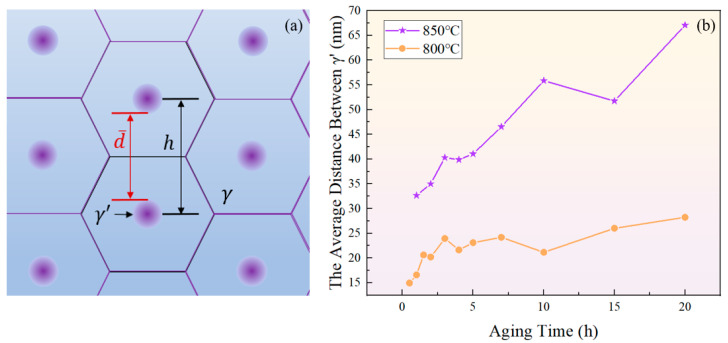
(**a**) Schematic diagram of close-packed particles; (**b**) variation of surface spacing between two adjacent $\gamma^{\prime }$ phases with aging time at 800 °C and 850 °C.

Assuming that the gaps between particles in unit area are arranged in a honeycomb pattern, as depicted in Figure 18a, and the distance between the centers of two particles is h, the area of a regular hexagon can be calculated by $S_{RH}=\frac{\sqrt{3}}{2}h^{2}$. Further, it can be obtained based on the number of particles per unit area *N* that:

(12) $$h=\sqrt{\frac{2\sqrt{3}}{3}}\cdot \frac{1}{\sqrt{N}}$$

Hence, the average distance $\bar{d}$ between two $\gamma^{\prime }$ particles at a certain moment is represented as:

(13) $$\bar{d}=\sqrt{\frac{2\sqrt{3}}{3}}\cdot \frac{1}{\sqrt{N}}-2\bar{r}$$

where $\bar{r}$ is the average diameter of the $\gamma^{\prime }$ particles shown in Figure 5a.

As shown in Figure 18b, the increase in the average distance between $\gamma^{\prime }$ particles indicates a prolonged time required for solute atoms to reach equilibrium through diffusion, which corresponds to increased intervals between the extrema curves of the total content of the $\gamma^{\prime }$ phase. Previous research has shown that the redissolution of the $\gamma^{\prime }$ phase can lead to the precipitation of new $\gamma^{\prime }$ in oversaturated regions of elements [65]. The extensive occurrence of such a process during aging for 4 h and 10 h at 850 °C, and for 2 h, 4 h, and 10 h at 800 °C brought about a decrease in particle spacing.

## 5. Conclusions

(1) The resistivity exhibits fluctuation over aging time and is negatively correlated with the area fraction of the $\gamma^{\prime }$ phase. This indicates that the solute concentration in the matrix is the primary factor for the resistivity change. The enrichment of solute atoms led to lattice distortion, facilitating the formation of short-range-ordered structures, which increase electron scattering and thus elevate the resistivity. Additionally, the precipitation and growth of $M_{23}C_{6}$ at grain boundaries also contributed to the gradual increase in resistivity over aging time.
(2) The $\gamma^{\prime }$ phase exhibited pronounced ripening during the aging process. The LSW ripening theory successfully predicted the size evolution of the $\gamma^{\prime }$ phase, and its coarsening activation energy was determined to be 277.59 kJ/mol, indicating that the coarsening of γ was primarily due to bulk diffusion of Al and Ti elements. Differences in the chemical potential of solute atoms in the matrix near the surfaces of differently sized $\gamma^{\prime }$ particles led to interactions between adjacent $\gamma^{\prime }$ particles. This resulted in the redissolution of small-sized $\gamma^{\prime }$ particles and the growth of large-sized $\gamma^{\prime }$ particles. Since the growth rate was not consistent with the dissolution rate, fluctuations appeared in the area fraction of the $\gamma^{\prime }$ phase.
(3) By connecting the inflection points of the resistivity change curves at different temperatures, the extrema curves of the total content of the $\gamma^{\prime }$ phase were obtained. These curves can be used to determine the total content changes of the $\gamma^{\prime }$ phase during aging at a specific temperature and time. This provides a new method for characterizing the fluctuation of the total content of the $\gamma^{\prime }$ phase.

## Figures and Tables

**Table 1:** Chemical composition of the novel nickel–iron-based alloy (wt.%).

Element	Fe	Ni	Cr	Al	Ti	Co	Mo	C + Si + Mn + B + Nb + W
wt.%	42.82	Bal.	16.1	1.4	2.0	2.0	0.5	0.4

## Data Availability

The raw data supporting the conclusions of this article will be made available by the authors on request.
